# Profile of Medicinal Plants Traditionally Used for the Treatment of Skin Burns

**DOI:** 10.1155/2022/3436665

**Published:** 2022-06-06

**Authors:** Hanae Naceiri Mrabti, Latifa Doudach, Mouna Mekkaoui, Zineb Khalil, Khouloud Harraqui, Fozia Fozia, Nidal Naceiri Mrabti, Mohamed El-Shazly, Amal Alotaibi, Riaz Ullah, Moulay El Abbes Faouzi, Abdelhakim Bouyahya

**Affiliations:** ^1^Laboratory of Pharmacology and Toxicology, Bio Pharmaceutical and Toxicological Analysis Research Team, Faculty of Medicine and Pharmacy, Mohammed V University in Rabat, BP 6203, Rabat, Morocco; ^2^Biomedical Engineering Department, National School of Arts and Crafts Rabat (ENSAM), Mohammed V University in Rabat, BP 6203, Rabat, Morocco; ^3^Pharmacodynamics Research Team, Laboratory of Pharmacology and Toxicology, Faculty of Medicine and Pharmacy, Mohammed V University in Rabat, Rabat, Morocco; ^4^Laboratory of Medicinal Chemistry, Drug Sciences Research Center, Faculty of Medicine and Pharmacy, Mohammed V University in Rabat, BP 6203, Rabat, Morocco; ^5^Biology and Health Laboratory, Faculty of Sciences, Ibn Tofail University, P.O. Box 133, Kenitra 14000, Morocco; ^6^Biochemistry Department, Khyber Medical University Institute of Medical Sciences, Kohat, Khyber Pakhtunkhwa, Pakistan; ^7^Computer Chemistry and Modeling Team, Laboratory of Materials, Modeling and Environmental Engineering (LIMME), Faculty of Sciences Dhar El Mehraz, Sidi Mohamed Ben Abdellah University (USMBA), BP 1796, Atlas, 30000 Fez, Morocco; ^8^Department of Pharmacognosy, Faculty of Pharmacy, Ain Shams University, Cairo 11566, Egypt; ^9^Department of Pharmaceutical Biology, Faculty of Pharmacy and Biotechnology, German University in Cairo (GUC), Cairo 11835, Egypt; ^10^Department of Basic Science, College of Medicine, Princess Nourah Bint Abdulrahman University, P.O. Box 84428, Riyadh 11671, Saudi Arabia; ^11^Department of Pharmacognosy and MAPPRC, College of Pharmacy, King Saud University, Riyadh 11451, Saudi Arabia; ^12^Laboratory of Human Pathologies Biology, Department of Biology, Faculty of Sciences and Genomic Center of Human Pathologies, Faculty of Medicine and Pharmacy, Mohammed V University in Rabat, Rabat, Morocco

## Abstract

Moroccan folk healers use medicinal plants to treat several diseases including skin burns. The traditional knowledge of wound healing is not common among the general population. Only one ethnobotanical survey was carried out in Rabat, Morocco, to track the traditional use of medicinal plants in wound healing. Therefore, our report aimed to study the medicinal plants used in Taza region to treat wound healing. In total, 218 individuals participated in this survey. More than 40 medicinal plants belonging to 30 botanical families were cited as anti-burn remedies. The most commonly used medicinal plants were *Agave sisalana* L., *Nerium oleander* L., *Tetraclinis articulata* Benth., *Lawsonia inermis* L., *Artemisia herba-alba* Asso., and *Trigonella foenum-graecum* L. Most of the used medicinal plants belong to Asteraceae family. Comparing our results with the previous survey, we noted that twelve plants were reported for the first time as wound healing agents. The ethnomedicinal use showed that plants leaves are the most commonly used parts. Pulverization was the selected method of preparation. The direct application of powder to the burns was the most common way of treatment. Our study revealed, for the first time, the importance of medicinal plants to treat skin burns in Taza region. Our results could be considered as the stepping stone for creating a database of wound healing medicinal plants to promote scientific studies on these plants revealing their constituents and side effects.

## 1. Introduction

The skin is the largest organ in the body and provides many important functions including organ protection, percutaneous absorption, maintenance of body shape, fluid conservation, temperature control, and sensory and disease control [1]. Skin diseases are a major health problem worldwide. Skin burns are among the most common skin traumas in all age groups. Burns are defined as the partial or total destruction of the skin covering or underlying tissue by a thermal, electrical, or chemical agent or by ionizing radiation [2]. Most burn treatments start with a topical application of a soothing, protective, and anti-infective medication to prevent infection [3]. Since Antiquity, many plants were used by indigenous peoples in different regions of the world for the treatment of wounds and burns. Usually, ointments formulated from different medicinal plants have been used as curative agents due to their widespread diversity of medicinal ingredients such as terpenoids, tannins, alkaloids, flavonoids, essential oils, phenolic compounds, saponins, and fatty acids which exhibit abundant pharmacological potential like anticancer, antidiabetic, and antimicrobial effects as well as cosmetic properties [4–7]. Besides, it has been discovered currently that some bioactive constituents improve the curative progression of burns [8–10]. These phytoconstituents are not only inexpensive but also harmless. The occurrence of many life-supporting phytoconstituents in plants has prompted scientists to scientifically evaluate these plants for potential wound healing properties [11]. The development of natural resources is a goal that is becoming more and more important in many countries. Medicinal plants are used for treatment purposes of infections. These plants are subsidized as a foundation of stimulation for new beneficial phytoconstituents as well as color, flavor, and taste of food [12–17]. The WHO indorses the assessment of the efficacy and of plant-based medications to standardize their usage and integrate them into conventional healthcare systems [18]. Traditional medicinal practices differ greatly from country to country and region to region. They are influenced by many factors including culture, history, generational anecdotes, and local healers' philosophies. According to the WHO, nearly 80% of the developing countries' population use traditional medicine as the primary source of therapy [18]. In Morocco, medicinal plants inhabit a significant room in medicinal systems and play an important part in the national economy [19], and numerous investigations showed recently remarkable results for future pharmaceutical applications [20–25]. Morocco is one of the Mediterranean countries with a long tradition of cultivating and using medicinal plants. In the northeastern part of Morocco, the use of traditional medicine is widespread, and several herbal remedies used individually or in combination with other agents are recommended for the treatment of burns. Despite the widespread use of medicinal plants, the scientific categorization of the local knowledge describing how to prescribe these plants is threatened with loss. The knowledge is usually transferred from generation to generation verbally, which affects the accuracy and spread of information in local populations. One scientific approach to tackle this problem is to conduct surveys among certain populations and collect as much data as possible. These data are then categorized, analyzed, compared, scrutinized, and presented to the scientific and local communities in a clear reliable format to be preserved in a proper way for future generations. The widespread use of medicinal plants to treat skin burns in Morocco encouraged us to conduct surveys among local populations to understand and preserve local practices. After searching the literature, we found no ethnopharmacological surveys conducted on the use of medicinal plants for the treatment of skin burns in the northeast part of Morocco [26]. The purpose of this study was to record and summarize the traditional practices of using medicinal plants in the treatment of burns in the region of Taza. The results of this survey will guide scientists in their future pharmacological and clinical work aiming to provide scientific evidence on the use of certain medicinal plants to treat skin burns.

## 2. Materials and Methods

### 2.1. Description of the Study Area

**Figure 1:**
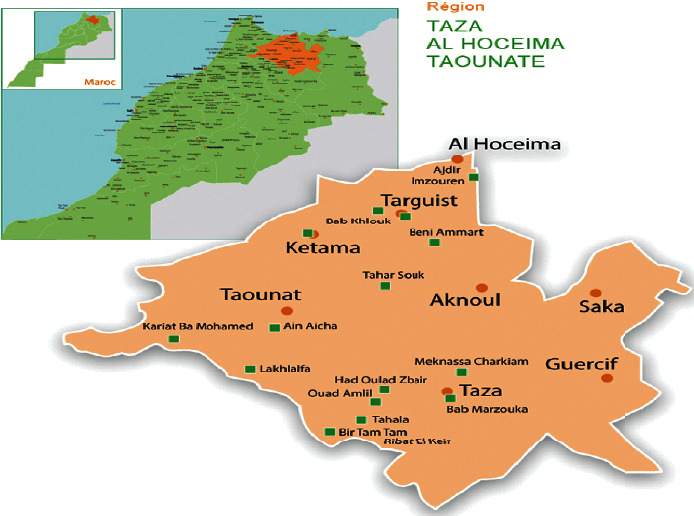
Map of the study area.

This study was carried out in the province of Taza. This city is administratively part of the Region of Fes-Meknes. Taza is a town located in the northeast of Morocco in the Taza corridor, a mountain pass where the Rif and Middle Atlas Mountains meet. The city is the capital of its province. It is located 220 km west of Oujda and 316 km east of Rabat (Figure 1). This city covers an area of 37 km^2^ with a population of 152,678 inhabitants in 2020. This city was selected because we observed the widespread use of medicinal plants to treat wound healing. Moreover, no ethnopharmacological study was conducted in this region to collect information about the use of medicinal plants in wound healing.

### 2.2. Collection of Data

The study was carried out from January 2021 to April 2021. Ethnobotanical knowledge was obtained through semi-assembled discussions. Interviews were carried out, and plant names in local dialect were recorded when cited. A total of 218 participants were interviewed for this survey (Table 1). The interviews were planned to register data about plants used for healing purposes of skin burns and their homegrown names, methods of preparation, parts of the plant used, drug management, and demographic characteristics of the study participants (Table 1).

## 3. Results and Discussion

### 3.1. Sociodemographic Characteristics of Herbalists

Ethnobotanical surveys require questioning herbalists, traditional healers, and/or people with long experience in medicinal plants. The data obtained from questionnaires highlight the sociodemographic characteristics of herbalists and traditional healers. In this work, the number of participants was 218 individuals practicing traditional medicine in Taza. The age of these traditional practitioners ranged between 30 and 90 years with a high rate for the age group of 50 and 70 years (Table 1). It was also noted that women actively participated in this survey (83.49%) compared with men (16.51%). This is in line with previous studies carried out in Morocco, where women were more interested in traditional medicine [27, 28]. Unfortunately, most participants did not have formal education (75.69%). However, only 16.97% have primary level, 5.5% have secondary level, and 1.83% have university level education. Most participants declared that their knowledge was inherited from older family members (92.66%), while 7.34% acquired their knowledge from traditional practices (traditional initiation). The transmission of this traditional knowledge was carried out exclusively by families, and this can lead to the disappearance of certain information (plants not yet known) because new generations became less interested in traditional knowledge.

### 3.2. The Diversity of Medicinal Plants Used to Treat Burns

The survey revealed the importance of medicinal plants in treating burns. As listed in Table 2, [47] medicinal plants were used to treat burns. These species belong to 30 different botanical families. Different species were recognized by their vernacular names, which showed the diversity of the regional language, and the information was collected by analyzing and categorizing the location of the population. In our previous work, we showed that the region of Taza is rich in medicinal plants such as *Agave sisalana* L., *Nerium oleander* L., *Tetraclinis articulata* Benth., *Lawsonia inermis* L., *Artemisia herba-alba* Asso., and *Trigonella foenum-graecum* L. which are used not only for skin burns but also for other pathologies such as diabetes and diseases related to the digestive system [27]. Despite the richness of Taza with medicinal plants, a comparison of the diversity of medicinal plants between Taza and other regions was never conducted. Only Salhi et al. [3] carried out a study including six cities in the Rabat region (Rabat, Sale, Temara, Skhirat, Khemisset, and Tiflet). In the study of Salhi et al. [3], thirty-six species belonging to 35 genera and 23 botanical families were identified.

### 3.3. Medicinal Plants Previously Reported for Dermatology Uses

The only work that was reported on the anti-burn properties of Moroccan medicinal plants was that of [3] in the region of Rabat. Other surveys done in different Moroccan regions investigated the use of medicinal plants against different pathologies but did not focus on the use of medicinal plants against only burns. The results of the previous surveys are summarized in Table 2. A certain number of medicinal plants reported in our survey were cited in previous surveys and other ethnobotanical studies outside Morocco. However, several plants were newly cited in our survey such as *Narcissus poeticus, Ammi visnaga* (L.) Lam, *Nerium oleander* L., *Artemisia herba-alba* Asso., *Calendula arvensis* L., *Capparis spinosa* L., *Tetraclinis articulata* Benth., *Trigonella foenum-graecum* L., *Lupinus albus* L., *Mentha pulegium* L., *Eucalyptus globulus* Labill. (sp.), and *Alchemilla vulgaris*. These plants were not cited in any previous ethnopharmacological investigations and deserve more intensive pharmacological evaluation.

### 3.4. Other Pharmacological Activities of the Reported Medicinal Plants

Medicinal plants that showed anti-burn activity demonstrated other biological activities that need to be validated by extensive research. In our work, we carried out bibliographical research to see if the mentioned medicinal plants were subjected to experimental investigation focusing on the anti-burn activity. Certain plants were subjected to experimental investigation such as *Agave sisalana* L., *Conyza canadensis* L., and *Borago officinalis* L. (Table 2). Other species were not investigated for their wound healing activity including *Narcissus poeticus, Ammi visnaga* (L.) Lam, *Nerium oleander* L., *Artemisia herba-alba* Asso., *Calendula arvensis* L., *Capparis spinosa* L., *Tetraclinis articulata* Benth., *Trigonella foenum-graecum* L., *Lupinus albus* L., *Mentha pulegium* L., *Eucalyptus globulus* Labill., and *Alchemilla vulgaris*. More ethnomedicinal surveys should be carried out to preserve information on the use of medicinal plants as anti-burn agents in other regions of Morocco. Thorough medicinal surveys will allow the identification of potential plants and isolation of biologically active agents as drug leads.

### 3.5. Ethnic Medicinal Characteristics: The Used Parts of Plants, Methods of Preparation, and Administration

From the above, it is important to explore the uses of medicinal plants because they are used for the treatment of different infections. World Health Organization reports that various plant fractions and their dynamic constituents are utilized as traditional medicines of the world population [71–73].

**Figure 2:**
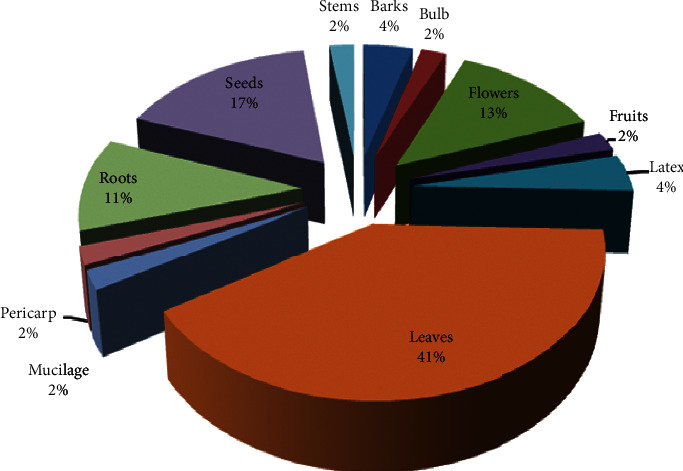
The plant parts used.

Our data showed that the leaves were the most used parts (41%) of medicinal plants, followed by seeds (17%), flowers (13%), roots (11%), bark and latex (4% each), fruits, bulbs, stems, pericarp, and mucilage (2% each) (Figure 2). Our results were similar to the only work carried out in Morocco by Salhi et al. [3] on plants used against skin burns. Other work carried out in Morocco on medicinal plants against different pathologies showed that the leaves were the most commonly used parts [19, 27, 28, 30, 68]. The results demonstrated that the powder was the main and simplest traditional application method used in the treatment of skin burns either alone or in combination with adjuvants such as honey, olive oil, and rose oil. Similar results were reported by Salhi et al. [3].

## 4. Conclusion and Perspectives

We surveyed and summarized the medicinal plants used to treat skin diseases in the Taza region. The traditional knowledge demonstrated in this work showed that ethnobotanical surveys can play a decisive role in screening plants with biological properties such as wound healing activity. The results of our work can guide scientists in their selection of plants to be studied experimentally to treat burns. Other surveys should also be carried out in other regions of Morocco to highlight all the medicinal species treating skin burns in Moroccan folk medicine and thus preserve such valuable knowledge for future generations. In addition, medicinal plants that revealed healing effects in our study should be studied for their *in vivo* properties. In addition, powders of these species could be prepared as formulations for their applications against skin burns.

## Figures and Tables

**Table 1:** Sociodemographic characteristics and experience of herbalists.

Characteristics	Number of informants (*n*)	Frequency (%)
Age (years)		
30–50	65	29.81
50–70	91	41.74
70–90	62	28.44
Total	218	100

Gender		
Male	36	16.51
Female	182	83.49
Total	218	100

Education		
None	165	75.69
Primary	37	16.97
Secondary	12	5.50
University	4	1.83
Total	218	100

Origin of knowledge		
Family heritage	202	92.66
Traditional initiation	16	7.34

Total	218	100

**Table 2:** Medicinal plants used to treat skin burns.

Family name	Plant species [voucher no.]	Vernacular name	Parts used	Method of preparation and application	FC	Recorded literature for ethnomedicinal uses in Morocco	Recorded literature for ethnomedicinal uses worldwide
Agavaceae	*Agave sisalana* L. [RAB1371]	Sabra	Mucilage	The mucilaginous extract of the fresh leaves is applied as a poultice on burns.	0.98	Skin diseases [29] and eczema [30]	ND

Amaryllidaceae	*Narcissus poeticus* [RAB1371]	Narjis	Flowers	The flower powder is mixed with olive oil and applied as a poultice on burns.	0.44	ND	ND

Anacardiaceae	*Pistacia atlantica* Desf. [RAB1372]	Drou	Barks	The bark powder is sprinkled on burns.	0.61	Diabetes [31, 32], allergy, digestive ailments, cardiovascular diseases, diabetes [33], digestive ailments, respiratory ailments, urogenital affections [34, 35], abdominal colic [36], obesity, and hair care [37]	ND

Apiaceae	*Ammi visnaga* L. [RAB12423]	Bachnikha	Fruits	The powder of the fruits is sprinkled on burns.	0.38	ND	ND
	*Daucus carota* L. [RAB109243]	Khizzu	Roots	The juice extracted from the roots is used as a compress to clean burns.	0.22	Diabetes [32, 38], stomach disorders [29], helminthiasis [39], urinary infections [40, 41], and burns [3, 30]	Burns, skin toner [42], eczema [43]

Apocynaceae	*Nerium oleander* L. [RAB18820]	Defla	Leaves	The powder of leaves is sprinkled on burns.	0.87	ND	ND

Aristolochiaceae	*Aristolochia paucinervis* [RAB18821]	Baraztam	Leaves	The leaf powder is mixed with olive oil and applied as a poultice on burns.	0.54	Urogenital affections [34], dermatological and digestive ailments, and rheumatology [44]	ND

Asteraceae	*Artemisia herba-alba* Asso., [RAB109244]	Chih	Leaves	The leaf powder is mixed with honey and applied as a poultice on burns.	0.71	ND	ND
	*Insula viscosa* (L.) Ait. [RAB109244]	Terklan	Roots	The powder of roots is sprinkled on burns.	0.11	Diabetes, digestive system, cancer, and skin diseases [29]	ND

	*Conyza canadensis* L. [RAB109244]	Elatassa	Leaves	The leaf powder is mixed with olive oil and applied as a poultice on burns.	0.19	Skin diseases [29]	ND
	*Cynara humilis* [RAB79161]	Timta	Roots	The powder of roots is sprinkled on burns.	0.12	Burns [36]	ND
	*Atractylis resinifera* L. [RAB79162]	Addad	Roots	The root powder is mixed with olive oil and applied as a poultice on burns.	0.27	Skin abscesses and warts [30, 39]	ND
	*Calendula arvensis* L. [RAB14312]	Zwiwl	Flowers	The flower powder is mixed with olive oil and applied as a poultice on burns.	0.18	ND	ND
	*Dittrichia viscosa* (L.) Greuter [RAB14314]	Magraman	Leaves	The powder of leaves is sprinkled directly on burns.	0.33	Diabetes [32], digestive system [40], bronchitis [45] burns, wounds, abscesses [30], urogenital affections, fever, rheumatology, and digestive system [34]	ND
	*Matricaria chamomilla* [RAB15115]	Babounj	Flowers	The flower powder is mixed with olive oil and applied as a poultice on burns.	0.46	Diabetes [19, 32, 38, 45], digestive system, dermocosmetology [33, 35], antineuralgic, febrifuge, antispastic of digestive organs, emmenagogue, reduced allergy [37, 46], neuralgia, anxiety, insomnia, spasmolytic, and wounds [39, 40]	ND

Brassicaceae	*Lepidium sativum* L. [RAB14317]	Habb rchad	Seeds	The powder of the seeds is sprinkled on the burns.	0.73	Diabetes [19, 38, 45], chronic diseases [47], cardiovascular diseases [33], bronchitis, cold, cough [45], eczema, skin ulcers and warts, stomach aches, anemia [30], and asthma [36, 48]	ND

Boraginaceae	*Borago officinalis* L. [RAB14318]	Lsan tour	Leaves	The latex extracted from the leaves is applied as a poultice to the burns.	0.8	Diabetes [45], anti-inflammatory, nervousness, respiratory canals, skin diseases [29, 40], colds, fever, diuretic, and laxative [39]	ND

Cannabaceae	*Cannabis sativa* L. [RAB14319]	Kif	Leaves	Burning leaves are sprinkled on burns.	0.16	Narcotics, skin diseases, and hair strengthening [30, 41, 49, 50] [40]	ND

Capparaceae	*Capparis spinosa* L. [RAB97161]	Lekbar	Seeds	The powder of the seeds is sprinkled on burns.	0.28	ND	ND

Cistaceae	*Cistus monspeliensis* [RAB97162]	Chteppa	Leaves	The leaves are applied as a poultice to burns.	0.29	Wounds [49], respiratory diseases [45], and diabetes [51]	ND

Cupressaceae	*Tetraclinis articulata Benth*. [RAB18717]	Al'Araâr	Leaves	The powder of the leaves is sprinkled directly on the burns.	0.91	ND	ND

Euphorbiaceae	*Euphorbia* sp. [RAB18717]	Loubina	Latex	The latex extracted is applied as a poultice to burns.	0.2	Skin diseases and cytotoxicity [29, 30]	ND
	*Ricinus communis* [RAB18718]	Alkharwaa	Seeds	The powder of the seeds is sprinkled on burns.	0.13	Toxic [29], diabetes [52], digestive system [40], skin diseases [30, 35], headache [36], antipyretic, rheumatism, diarrhea, laxative [53], fever [54], and hair care [44, 51]	ND

Fabaceae	*Trigonella foenum-graecum* L. [RAB24117]	Lhelba	Seeds	The seed powder is mixed with rose oil and applied as a poultice on burns.	0.89	ND	ND
	*Lupinus albus* L., [RAB21118]	Termes	Seeds	The seed powder is mixed with olive oil and applied as a poultice on the burns.	0.51	ND	ND

Gentianaceae	*Centaurium erythraea* [RAB22415]	Kassat lahya	Flowers	The flower powder is sprinkled on burns.	0.22	Diabetes [38, 45, 55], skin diseases [49], allergy, increasing energy [33], digestive system, and kidney diseases [29, 44]	ND

Juncaceae	*Juncus acutus* L. [RAB47241]	Assmar	Latex	The extracted latex is applied as a poultice to burns.	0.14	Skin diseases [29].	ND

Lamiaceae	*Marrubium vulgare* L. [RAB47249]	Mriwt	Leaves	The leaf powder is mixed with olive oil and applied as a poultice on burns.	0.26	ND	ND
	*Mentha pulegium* L.	Flio	Leaves	The leaf powder is mixed with olive oil and applied as a poultice on burns.	0.39	ND	ND
	*Salvia verbenaca* [RAB109218]	Khiyyata	Leaves	The leaf powder is sprinkled on burns.	0.46	Cardiac disease, diabetes [45], respiratory and rheumatologic conditions [34], abdominal colic, cold, fever [36], and wounds [56]	Wound healing [57], wounds [58], antiseptic on wounds [42], skin inflammations, and bacterial infections of the skin [59]
	*Lavandula angustifolia* [RAB109229]	Lakhzama	Leaves	The leaf powder is mixed with olive oil and applied as a poultice on burns.	0.29	Diabetes [38], digestive system [40, 44], and burns [3]	Dermatitis, furuncle, abscess, wart [60], wound healing [61].

Leguminosae	*Retama raetam* (Forssk.) [RAB109231]	Rtem	Leaves	The leaf powder is mixed with honey and sprinkled on the burns.	0.32	Skin diseases, toxic [29], and diabetes [32]	ND

Liliaceae	*Urginea maritima* L. [RAB23142]	Bessal lanssal	Bulb	The bulbs triturated in butter are applied as a poultice to burns.	0.11	Cattle ailments, skin disorders [29], abscesses, alopecia, sedative, hemorrhoids [30], and digestive system [44]	ND

Linaceae	*Linum usitatissimum* [RAB109227]	Zariat lktan	Seeds	The seed powder is sprinkled on burns.	0.78	Diabetes [30, 62], asthma [47], renal disease [50], laxative, diuretic, and vermifuge [39]	Skin burns [63], wound healing [43], dermatological infections [58, 64], healing skin [42]

Lythraceae	*Lawsonia inermis* L. [RAB109226]	Lehana	Leaves	The leaf powder is sprinkled on burns.	0.112	Wounds, dermatoses [56], burns, eczema, mycosis, boils, abscesses, chapped skin, antiseptic, healing wounds [3], eczema [30], diabetes [32], and dermocosmetology [41]	Wound healing [65], eczema [66], wrinkled skin, abscess [67]
	*Punica granatum* [RAB109230]	Raman	Pericarp	Fruit pericarp powder is mixed with olive oil and applied as a poultice on burns.	0.54	Diabetes [30, 47, 50], stomach disorders [49], diabetes, digestive system [29, 41], eczema [39], and wounds [36].	ND

Myrtaceae	*Eucalyptus globulus* Labill. [RAB9318]	Al' Kalitouss	Leaves	The leaf powder is sprinkled on burns.	0.16	ND	ND
	*Myrtus communis* L. [RAB49621]	Arraihan	Leaves	The leaf powder is mixed with rose oil and applied as a poultice on burns.	0.5	Diabetes [30, 45], cardiac disease, hypertension, [49], cardiac weakness, and digestive system [29]	ND

Oleaceae	*Olea europaea* L. *var*. *oleaster* [RAB51120]	Zabouj	Leaves	The essential oil is applied as a poultice on burns.	0.25	Nervousness and anthelmintic [29]	ND

Papaveraceae	*Papaver rhoeas* L. [RAB51218]	Belaaman	Flowers	The flower powder is mixed with honey and applied as a poultice on burns.	0.9	Urogenital affections, hair care [34], against fever, sleep troubles and asthma [68], cold, antimicrobial [37], asthma, cough, improving breath, sedative, skin diseases [36], fever [30], and diabetes [19]	ND

Pinaceae	*Pinus halepensis* L. [RAB93519]	Taydâ	Barks	The bark powder is mixed with olive oil and applied as a poultice on burns.	0.37	Against toothache [49] and tuberculosis [39, 41]	ND

Plantaginaceae	*Plantago coronopus* L. [RAB109241]	Massassa	Stems	The fruit stems are sprinkled on the burns.	0.12	Abscess and skin diseases [29]	ND

Rosaceae	*Prunus armeniaca* L. [RAB41111]	Machmach	Seeds	The seed powder is mixed with olive oil and applied as a poultice on burns.	0.9	Diabetes [30], face care [39], and aphrodisiac [41]	ND

Rosaceae	*Rosa centifolia* L. [RAB41113]	Lward	Flowers	The essential oil is applied as a poultice on burns.	0.52	Cosmetic and skin face [29]	ND

Rosaceae	*Alchemilla vulgaris* [RAB41114]	Gdam sbaâ	Leaves	The powder of leaves is sprinkled on the burns.	0.17	ND	ND

Zingiberaceae	*Curcuma longa* L. [RAB41118]	Lkharkoum	Roots	The root powder is mixed with honey and applied as a poultice on burns.	0.89	As a condiment, tonic, calefacient, and digestive [39], digestive stimulant, for blood diseases, and against amnesia [41]	ND

Zygophyllaceae	*Peganum harmala* L. [RAB41116]	Lharmel	Seeds	The powder of seeds is sprinkled on the burns.	0.25	Induce abortion [69], diabetes [32, 38, 69, 70], hair care [30, 53, 56], spasmolytic and anthelmintic [39], toxic, sedative, nervous system disorders, rheumatism, decrease lipids [37], abdominal colic, induce abortion, anti‑spasmodic, cold, diarrhea, eczema, hemorrhoids, jaundice, rheumatism, women sterility, and wounds [36]	ND

ND : not determined FC explained in the manuscript : Frequency of Citation.

## Data Availability

All the available data used to support the findings of the study are included within the article.
